# Systemic Lupus Erythematosus and Secondary Antiphospholipid Syndrome after Thymectomy for Myasthenia Gravis - A Case Report

**DOI:** 10.3889/oamjms.2015.096

**Published:** 2015-08-26

**Authors:** Rada Miskovic, Aleksandra Plavsic, Aleksandra Peric-Popadic, Sanvila Raskovic, Mirjana Bogic

**Affiliations:** *Clinical Center of Serbia, Clinic for Allergology and Immunology, Belgrade, Serbia*

**Keywords:** systemic lupus erythematosus, antiphospholipid syndrome, autoimmune diseases, myasthenia gravis, thymectomy

## Abstract

**INTRODUCTION:**

Systemic lupus erythematosus (SLE) and myasthenia gravis (MG) are autoimmune diseases that show some similarities: a higher incidence in young women, relapsing-remitting course and positive anti-nuclear antibodies (ANA). However, they are two different clinical syndromes, which can coexist or precede each other. Thymectomy is a therapeutic option for patients with severe MG or thymoma. There are many cases of SLE after thymectomy described in the literature, so the question arises whether thymectomy predisposes patients to SLE and what are imunopathogenetic mechanisms behind this process.

**CASE REPORT:**

We report a case of a patient who was diagnosed with SLE and secondary antiphospholipid syndrome (APS) 28 years after thymectomy for MG. Clinical picture of SLE was characterized by cutaneous and articular manifestations, polyserositis, lupus nephritis and immunological parameters showed positive ANA, anti-ds-DNA, excessive consumption of complement components, positive cryoglobulins. Clinical and laboratory immunological parameters for the diagnosis of secondary APS where also present. The patient was initially treated with glucocorticoids followed by mycophenolate mofetil. During one year follow-up patient was in a stable remission of SLE.

**CONCLUSION:**

Thymectomy for MG may predispose SLE development in some patients. Further studies are needed to better understand the connection between these two autoimmune diseases.

## Introduction

Systemic lupus erythematosus (SLE) is a systemic autoimmune disease characterized by multiorgan dysfunction, production of numerous autoantibodies and higher incidence in women of childbearing age. Myasthenia gravis (MG) is an organ-specific autoimmune disease characterized by dysfunction of the neuromuscular junction mediated by autoantibodies directed to: the nicotinic acetylcholine receptor (nAChR), the muscle specific tyrosine kinase (MuSK) or the low-density lipoproteinreceptor-related protein 4 (LRP4) with the consequent muscle weakness [1, 2]. In regard to the clinical picture, type of autoantibodies present, age of the patient at the diagnosis and thymus pathology, patients with MG can be classified into different subgroups. Therapeutic modalities include use of immunosuppressive drugs, supportive therapy and thymectomy [3]. Thymectomy is a therapeutic option for patients with non-thymomatous MG, as well for patients with thymoma regardless of the severity of MG (3).

The literature describes cases of SLE after thymectomy, but pathogenetic mechanisms that lead to this phenomenon are not clear. We present a case of a patient who was diagnosed with SLE and secondary APS 28 years after thymectomy performed for MG.

## Case report

A 48 years old female patient was admitted to the Clinic for Allergy and Immunology under suspicion of systemic connective tissue disease. The main complaints were marked fatigue, photosensitivity, hives, swelling and pain in the small joints of hands, wrists and ankles, Raynaud’s phenomenon. About seven months before admission the patient was treated at the regional hospital for pulmonary embolism and exudative pericarditis. Three years before admission she was examined by a hematologist due to generalized lymphadenopathy, and after a detailed examination a lymphoproliferative disorder was ruled out. Her past medical history revealed that she underwent thymectomy for generalized form of MG 28 years ago, after which a complete long-term remission was achived.

Physical examination on admission showed hives, generalized lymphadenopathy, cyanotic lips, spontaneous Raynaud’s phenomenon on fingers, acute synovitis of proximal interphalanegal and metacarpophalangeal joints and wrists, and perimaleolar pitting edema. Other physical findings were normal. Laboratory studies showed marked inflammatory syndrome, elevated total serum proteins, hypoalbuminemia, elevated serum alkaline phosphatase, G-glutamyl transferase, lactate dehydrogenase. Granulated cylinders were seen in the urine sediment, with 24h-proteinuria of nephrotic range and slightly reduced creatinine clearance. The result of blood gas analysis was normal. Immunoserological analysis showed positive ANA on a substrate of HEp2 cells (indirect immunofluorescens) in a dilution higher than 1: 640 homogeneous type of staining, positive anti-dsDNA and anti-cardiolipin antibodies (both IgG and IgM type), presence of cryoglobulins, with marked signs of complement activation. Serum immunoglobulins levels were extremely high. Direct Coombs test was positive, but there were no other laboratory or clinical signs of active haemolysis.

Table 1 shows the results of laboratory and immunological analysis at the diagnosis of SLE and secondary APS.

**Table 1:** Laboratory and immunoserological analysis of the patient with SLE and APS after thymectomy for MG

Analysis	Normal values	Finding
ESR (1h) (mm/h)	2 - 10	68
C-reactive protein (mg/l)	0 - 5	30
Leukocytes (x 10^9^/l)	3.5 – 10	8.0
Neutrophils (x 10^9^/l)	2.1 – 6.5	4.0
Hemoglobin (g/l)	122 – 157	142
Erythrocytes (x 10^12^/l)	3.86 – 5.08	4.9
MCV (fl)	83 - 97	88
Urea (mmol/l)	2.8 – 7.2	6.7
Creatinine (µmol/l)	58 – 96	92
Aspartate aminotransferase (U/l)	0 – 31	30
Alanine aminotransferase (U/l)	0 - 34	28
Total serum proteins (g/l)	66 - 83	84
Albumines (g/l)	35 - 53	29
G-glutamyl-transferase (U/l)	4- 32	93
Lactate dehydrogenase (U/l)	0 - 247	729
Alkaline phosphatase (U/l)	30 - 120	214
Creatinine clearance (ml/min)	87 -107	68.7
Urine cylinders (cells/HPF)	0 -2 hyaline cylinders	3-4 granulated cylinders
24h-proteinuria	0.05 – 0.150	6.8 g/24h
ANA Hep2 – IIF	negative	>1:640 homogenous type of staining
Anti-ds-DNA	negative	1:40
Anticardiolipin Ab IgG (ELISA) (GPL-U/ml)	0 – 12	47.1
Anticardiolipin Ab IgM (ELISA) (MPL-U/ml)	0 - 12	93.4
Cryoglobulins	negative	+++
Complement C3 (g/l)	0.9 – 2.07	1.17
Complement C4 (g/l)	0.12 – 0.36	0.08
IgG (g/l)	6.5 – 16	> 30
IgM (g/l)	0.5 – 3	> 6
IgA (g/l)	0.4 – 3.5	2.82
Coombs test (anti-IgG)	negative	++

ESR – erythrocyte sedimentation rate; MCV – mean corpuscular volume; Ig – immunoglobulin.

A minor pleural effusion on the right side and exudative pericarditis were seen on the chest radiography. Lupus Band Test (LBT) was positive. Abdominal sonography showed an enlarged liver (166 cm). Echocardiography demonstrated pronounced enlargement of the right heart, with 4 + tricuspid regurgitation, pericardial effusion around the entire heart, enlarged pulmonary vein and paradoxical movement of the septum, left atrium collapsed during diastole, the pressure in the right ventricle was estimated at 100 mm Hg. Multislice CT pulmonary angiography was normal.

The diagnosis of SLE was made based on the following criteria of the American College of Rheumatology (6/11): photosensitivity, polyarthritis, polyserositis (pericardial and pleural effusion), renal lesions (abnormal urine sediment, nephrotic rank proteinuria), positive ANA, anti-dsDNA and anti-cardiolipin antibodies. The diagnosis of secondary antiphospholipid syndrome was based on the Sapporo criteria: pulmonary embolism and positive anticardiolipin antibody IgG and IgM type on two occasions for a period longer than 12 weeks. The patient was treated using glucocorticoid (initialy methyl-prednisolon 1 mg/kg body weight, followed by three ’pulse’ doses of methyl-prednisolone, each 1,000 mg) and anticoagulant therapy with favorable clinical and laboratory response. In the further course, a renal biopsy was performed and pathohistological examination revealed diffuse proliferative glomerulonephritis. Mycophenolate mofetil therapy was introduced at a dose of 2 grams orally with oral prednisone therapy. During the one-year follow-up period patient was in a stable remission of SLE.

## Discussion

Systemic lupus erythematosus and myasthenia gravis are autoimmune diseases that share some similarities. They occur more often in women, the clinical course is characterized by periods of exacerbation and remission and both share positivity for antinuclear antibodies [4]. It is known that patients who are affected by one autoimmune disease are at increased risk of developing the second one. The frequency of second autoimmune disease is 13 – 22 % in patients with MG [5-8]. Large population based nested case-control study of Swedish authors, analyzing 2045 MG cases, has showed the presence of other autoimmune diseases in 449 (22.0%) patients, most commonly hypothyroidism, rheumatoid arthritis, type I diabetes, and psoriasis. The patients with MG had higher prevalence of other autoimmune disease compared to a control group of general population [8]. On the other hand, the development of MG in SLE patients is rarely reported. In a study of Canadian authors who followed 380 patients with SLE over a period of 9 years, the diagnosis of MG was established only in one patient [9]. The possible explanation may be that in SLE patients, the muscle weakness may be considered as a complication of glucocorticoid therapy (glucocorticoid myopathy) rather than a symptom of MG. Therefore, MG should be included in the differential diagnosis of SLE patients with muscle weakness [7]. These data suggest that SLE and MG are two distinct autoimmune diseases that may coexist, but the immunopathogenic basis of this association is not yet clear. It is possible that they both share certain common genetic, immunological and environmental factors that may be important in the pathogenesis of both disorders [10].

In the literature, the appearance of SLE after thymectomy for MG is documented mainly in case reports. In 1994 the group of Greek authors described a patient who was diagnosed with SLE 6 years after thymectomy that was performed for MG [11]. The French authors have concluded that development of SLE after thymectomy or thymomectomy may be more than a coincidence [12]. The SLE development after thymectomy for MG is described in two case reports of Korean authors [13]. In one patient, SLE has developed three months after thymectomy, while in the other patient 13 years after thymectomy. The main clinical manifestation of SLE in both patients was polyarthritis. The group of Italian authors have followed the long-term immunological effects after thymectomy in patients with MG [14]. There were 16 patients who underwent thymectomy at least 8 years before the study and the results were compared to a group of recently thymectomized and non-thymectomized patient with MG [14]. Patients who underwent thymectomy for more than 8 years had elevated serum IgG, IgM, anticardiolipin, anti- dsDNA antibodies and mild T - cell lymphopenia. After three years of follow-up, two of these 16 patients were diagnosed with systemic autoimmune diseases, one with SLE and other with undifferentiated connective tissue disease.

These data indicate that in some patients with MG who undergo thymectomy, SLE may develop over the years. The mechanisms behind this process are not completely understood, and the role of thymectomy itself is considered as the possible precipitating factor in the process of SLE development. The studies on experimental models suggest the development of autoimmune diseases after thymectomy [15, 16]. Thymus plays an important role in maintaining immune homeostasis through the processes of central tolerance that may be affected by pathological processes in it (hyperplasia, thymoma). It is possible that thymectomy can lead to imbalance of autoreactive and regulatory T cells and to the initiation of autoimmune processes due to the loss of central tolerance and excessive production of autoantibodies. Thymectomy may create conditions for the sequence of events that, in genetically predisposed individuals, can lead to development of SLE, in associations with environmental factors [13, 14].

In our case report, SLE was diagnosed 28 years after thymectomy that was performed for MG and and by which long-term remission of MG was achived. The main clinical SLE manifestations in our patient were diffuse proliferative lupus nephritis with nephrotic proteinuria. Our patient was also diagnosed with secondary APS. Systemic lupus erythematosus with lupus nephritis as the leading clinical manifestation after thymectomy is described in a case report by Lee and associates, although there was no pathohistological confirmation of lupus nephritis because the patient refused a kidney biopsy [10]. In other case reports in the literature, the most common clinical feature of SLE after thymectomy is polyarthritis, and to a lesser extent cytopenia, pleuritis, skin changes [13]. To our knowledge, the case of SLE with severe lupus nephritis and secondary APS after thymectomy for MG was not previously described in the literature.

Although the data from the literature suggest the coexistence of SLE and MG, the pathological mechanisms behind these association are not fully known, as well as potential triggers that can lead to the development of SLE in MG patients and vice versa. In this sense, Bhinder and associates have posed an intriguing question: are these two diseases part of the same autoimmune disease spectrum; which of these diseases is more likely to predispose to the development of the other and why? [17]. The literature data suggest that SLE can occur after thymectomy, so an interesting question emerges: is the thymectomy risk factor, rather than a possible predisposing factor for the SLE development in some patients? If so, whether patients with MG after thymectomy show some clinical characteristics that may indicate SLE diagnosis? How regularly they should be evaluated for the possible diagnosis of SLE?

In conclusion, the association between SLE and MG is complex. These two autoimmune diseases may precede one another or coexist. Thymectomy may be a precipitating factor that over time period can lead to the development of SLE in certain individuals. Therefore, in MG patients who have undergone thymectomy, any clinical and immune serological SLE suspicion should be carefully evaluated.
